# Avoiding gambling harm: An evidence-based set of safe gambling practices for consumers

**DOI:** 10.1371/journal.pone.0224083

**Published:** 2019-10-17

**Authors:** Nerilee Hing, Matthew Browne, Alex M. T. Russell, Matthew Rockloff, Vijay Rawat, Fiona Nicoll, Garry Smith

**Affiliations:** 1 Experimental Gambling Research Laboratory, School of Health, Medical and Applied Sciences, CQUniversity, Bundaberg, Queensland, Australia; 2 Experimental Gambling Research Laboratory, School of Health, Medical and Applied Sciences, CQUniversity, Sydney, New South Wales, Australia; 3 Experimental Gambling Research Laboratory, School of Health, Medical and Applied Sciences, CQUniversity, Melbourne, Victoria, Australia; 4 University of Alberta, Edmonton, Canada; University of Auckland, NEW ZEALAND

## Abstract

Prior studies have identified self-regulatory strategies that are infrequently used by problem-gamblers, but which might be protective if used. However, guidelines with evidence-based safe gambling practices (SGPs) that prevent gambling-related harm are lacking. This study aimed to: 1) identify a parsimonious set of evidence-based SGPs that best predict non-harmful gambling amongst gamblers who are otherwise most susceptible to experiencing gambling harm; 2) examine how widely are they used; and 3) assess whether their use differs by gambler characteristics. A sample of 1,174 regular gamblers in Alberta Canada completed an online survey measuring uptake of 43 potential SGPs, gambling harms and numerous risk factors for harmful gambling. Elastic net regression identified a sub-sample of 577 gamblers most susceptible to gambling harm and therefore most likely to benefit from the uptake of SGPs. A second elastic net predicted gambling harm scores in the sub-sample, using the SGPs as candidate predictors. Nine SGPs best predicted non-harmful gambling amongst this sub-sample. The behaviour most strongly associated with increased harm was using credit to gamble. The behaviour most strongly associated with reduced harm was ‘If I’m not having fun gambling, I stop’. These SGPs form the basis of evidence-based safe gambling guidelines which can be: 1) promoted to consumers, 2) form the basis of self-assessment tests, 3) used to measure safe gambling at a population level, and 4) inform supportive changes to policy and practice. The guidelines advise gamblers to: stop if they are not having fun, keep a household budget, keep a dedicated gambling budget, have a fixed amount they can spend, engage in other leisure activities, avoid gambling when upset or depressed, not use credit for gambling, avoid gambling to make money, and not think that strategies can help you win. These guidelines are a promising initiative to help reduce gambling-related harm.

## Introduction

A substantial minority of gamblers experience problem or at-risk gambling, leading to harmful consequences and reductions in health-related quality of life [1–3]. However, harm minimisation efforts to date have been criticised for focusing most attention on the small minority of gamblers with clinically significant gambling problems and for failing to reduce gambling-related harm [4–8]. Consistent with a public health approach, harm minimisation efforts need to extend beyond just reducing problem gambling, to also prevent harm amongst lower risk gamblers [9–12].

A widely used harm minimisation strategy is to advise all consumers to use various safe gambling practices (SGPs), but practices that are currently promoted are inconsistent and lack scientific evidence for their efficacy [13]. Some commonly promoted strategies have good face validity as they are a symptom of problem gambling and gambling disorder [14–15] (e.g., don’t chase your losses); however, others have conflicting research evidence for their effectiveness (e.g., don’t gamble alone [16]); or arguably provide little practical behavioural advice about how to implement the strategy (e.g., ensure your gambling does not cause harm for yourself or others). Evidence-based, directly actionable practices are needed to inform consumers how to keep their gambling safe.

Descriptive studies of practices used by different gambler risk groups dominate the literature on gambling self-regulatory strategies [17]. These studies have identified practices whose use is associated with lower-risk gambling, but have not yet examined them with gambling harm as, arguably, the most relevant outcome. Also, many are correlates of safer gambling, rather than behavioural strategies that could be implemented as a proactive measure. Wood and Griffiths [16] compared 1,484 ‘positive players’ and 209 problem gamblers. The former were more likely to engage in several non-gambling leisure activities; work out what they could afford to spend and set expenditure and time limits before gambling; and take only a predetermined amount of money and not take ATM cards when going to gambling venues. They placed less importance on feeling excited and feeling relaxed to enjoy a gambling session; and were less likely to gamble when bored, depressed or upset, and were less likely to gamble with friends and family. Amongst 860 regular gamblers, Hing, Sproston, Tran and Russell [18] found several practices associated with lower-risk gambling: setting a money limit before gambling; balancing gambling with other activities; being more motivated to gamble for pleasure and entertainment, and less for money, challenge or mood regulation. Wood, Wohl, Tabri and Philander [19] identified a pool of potential practices, using factor analysis in two samples of gamblers, to develop the Positive Play Scale. All four subscales of the Positive Play Scale (honesty and control, precommitment, personal responsibility, gambling literacy) correlated negatively with PGSI score [15].

The most comprehensive categorisation to date has identified 99 behaviour change strategies used by gamblers [20]. This study involving 489 gamblers, including 333 problem gamblers, factor analysed these strategies into 15 categories: cognitive, well-being, consumption control, behavioral substitution, financial management, urge management, self-monitoring, information seeking, spiritual, avoidance, social support, exclusion, planning, feedback, and limit finances. While differences in the use of these strategies was not reported by PGSI group, problem gamblers reported greater usefulness of all strategy categories than low and moderate risk gamblers, except for planning, limiting, finances, and consumption control for which no significant differences were found. An in-venue study with a 30-day follow-up recruited 104 participants from 11 gaming machine venues who completed the 30-item Gambling In-Venue Strategies Checklist [21]. Compared to problem gamblers, low risk/non-problem gamblers more frequently avoided chasing losses, set cues to keep track of time, used only the money brought into the venue, planned their spending in advance, and viewed gambling as entertainment. Numerous other studies have examined the uptake of more limited sets of self-regulatory strategies, generally finding less use amongst higher-risk gamblers (see [13]).

These studies provide useful insights into what SGPs lower-risk gamblers tend to use more than higher-risk gamblers. However, simply examining practices that correlate with PGSI group does not necessarily identify those that best protect against gambling harm. This is because lower-risk gamblers may not use some practices simply because they have little or no need to do so. For example, they would see little need to leave their bank cards at home as they can control their spending; whereas higher risk gamblers may be more likely to use this practice as a protective strategy. Use of this practice, while potentially protective, would therefore correlate with higher-risk rather than lower-risk gambling.

To avoid this confounding issue, we first identified a sample of gamblers who are susceptible to experiencing gambling harm based on the presence of known risk factors for gambling problems. We then compared the use of SGPs amongst those who either were, or were not, experiencing gambling harm. Unlike past research, we excluded people who are not susceptible to harm, since they might not use SGPs simply because they have no need. In recognition that SGPs that are currently promoted are inconsistent and lack scientific evidence for their efficacy in protecting against gambling-related harm, this study aims to: 1) identify a parsimonious set of evidence-based SGPs that best predict non-harmful gambling amongst gamblers who are most susceptible to experiencing gambling harm; 2) examine how widely are they used; and 3) assess whether their use differs by gambler characteristics.

## Methods

### Formative research

Our formative research identified the (potential) SGPs tested in this study. Several methods were used to generate a comprehensive pool of potential SGPs for testing, as described elsewhere [13]. This process commenced with a systematic literature search of major online databases and the grey literature using a wide range of relevant search terms (e.g., responsibl*, gambl*, self control, self limit*, self moderat*, self help, self regulat*, harm minimis*, harm reduc*, consumption and protect*), supplemented with a second round targeted search using search terms based on specific SGPs (e.g., precommit*, limit-set*, gambling budget, gambling motiv*). This search located 3,707 unique publications, with 96 directly relevant to safe gambling. Of these, 26 focused on safe gambling consumption and together identified 57 SGPs.

We then conducted a content analysis of gambling-related websites as these typically provide the most comprehensive consumer advice on safe gambling, and often replicate consumer information available in print (e.g., brochures, posters). Thirty websites were purposively selected as having a comprehensive suite of safe gambling information. Because this formative research was conducted in Australia, 25 Australian websites were analysed, as well as five international websites with particularly comprehensive information. They comprised six government, 10 industry and 14 help service websites. The content analysis identified 88 additional SGPs that these websites recommended for consumers.

Several SGPs from the literature review (57) and the content analysis (88) overlapped, and we collapsed these 145 practices to 61 items. A sample of 107 gambling research, treatment, training and policy professionals were then recruited by email from the research team’s professional contacts and from members of Gambling Issues International, a mailing list forum restricted to professionals who work with gambling issues. Using their professional judgment, the online survey asked these respondents to rate the importance of the 61 SGPs in helping people to gamble safely (on a 5-point scale from ‘not at all important’ to ‘extremely important’). They were also asked to identify any other SGPs that might be important, in an open-ended question. No other SGPs were identified that did not overlap with those already included in the survey. Ten items with mean ratings below the mid-point of the scale (‘moderately important’) were then discarded. The remaining 51 items were considered an appropriate foundation for the current research.

### Participants and procedure

A market research company, Qualtrics, recruited participants for an online survey in November and December 2017, and compensated them with points exchangeable for rewards according to their internal protocols. Inclusion criteria were: residing in Alberta Canada (location of the funding body); aged 18 years+; and at least monthly gambling (in aggregate) during the past 12 months on VLTs/slots, casino games, bingo, instant win tickets, race betting, sports betting, keno, eSports and fantasy sports. A total of 2,041 people started the survey, however 391 did not fully complete the survey and 476 failed one or more of the attention checks implemented throughout the survey. In total, 1,174 people completed the survey and met all inclusion criteria. We later subsampled from this group those most susceptible to gambling harm (*n* = 577). Table 1 presents demographic information for both samples.

**Table 1:** Demographic characteristics of the full sample and the subset.

Variable	Full sample (N = 1174) n (%)	Subset of gamblers (N = 577) n (%)
*Gender*		
Male	466 (39.7)	231 (40.0)
Female	705 (60.1)	343 (59.4)
Other	3 (0.3)	3 (0.5)
*Residence*		
Calgary	393 (33.5)	191 (33.1)
Edmonton	366 (31.2)	190 (32.9)
Regional town	169 (14.4)	86 (14.9)
Small town	174 (14.8)	82 (14.2)
Rural or remote location	72 (6.1)	28 (4.9)
*Language spoken at home*		
English	1142 (97.3)	558 (96.7)
French	4 (0.3)	1 (0.2)
Other	28 (2.4)	18 (3.1)
*Indigenous status*		
Non-Aboriginal	1100 (93.7)	523 (90.6)
First Nation	32 (2.7)	27 (4.7)
Métis	42 (3.6)	27 (4.7)
Inuk (Inuit)	0 (0.0)	0 (0.0)
*Marital status*		
Single/never married	302 (25.7)	199 (34.5)
Living with partner/defacto	164 (14.0)	93 (16.1)
Married	538 (45.8)	205 (35.5)
Divorced or separated	131 (11.2)	67 (11.6)
Widowed	39 (3.3)	13 (2.3)
*Country of birth*		
Canada	1051 (89.5)	507 (87.9)
Other	123 (10.5)	70 (12.1)
*Living arrangements*		
Live alone	233 (19.8)	126 (21.8)
Couple (no dependents)	379 (32.3)	161 (27.9)
Couple with at least one dependent child	237 (20.2)	95 (16.5)
Couple living with independent child(ren)	85 (7.2)	40 (6.9)
Single parent living with at least one dependent child	59 (5.0)	37 (6.4)
Single parent living with independent child(ren)	31 (2.6)	20 (3.5)
Share house with other adults	74 (6.3)	45 (7.8)
Live with parents	60 (5.1)	42 (7.3)
Other	16 (1.4)	11 (1.9)
*Highest level of education*		
Grade 8 or less	3 (0.3)	3 (0.5)
Some high school	76 (6.5)	59 (10.2)
High school diploma or equivalent	287 (24.4)	150 (26.0)
Registered Apprenticeship or other trades certificate or diploma	113 (9.6)	47 (8.1)
College, CEGEP or other non-university certificate or diploma	325 (27.7)	160 (27.7)
University certificate or diploma below bachelor’s level	75 (6.4)	41 (7.1)
Bachelor’s degree	235 (20.0)	98 (17)
Post graduate degree above bachelor’s level	60 (5.1)	19 (3.3)
*Work status*		
Work full-time	512 (43.6)	238 (41.2)
Work part-time or casual	165 (14.1)	99 (17.2)
Self-employed	89 (7.6)	41 (7.1)
Unemployed and looking for work	83 (7.1)	62 (10.7)
Full-time student	23 (2.0)	17 (2.9)
Full-time home duties	47 (4.0)	25 (4.3)
Retired	183 (15.6)	50 (8.7)
Sick or disability pension	58 (4.9)	40 (6.9)
Other	14 (1.2)	5 (0.9)
Occupation*		
Management	94 (8.0)	46 (8.0)
Business, finance and administration	92 (7.8)	42 (7.3)
Natural and applied sciences and related occupations	16 (1.4)	5 (0.9)
Health	89 (7.6)	34 (5.9)
Education, law and social, community and government services	80 (6.8)	31 (5.4)
Art, culture, recreation and sport	18 (1.5)	7 (1.2)
Sales and service	160 (13.6)	95 (16.5)
Trades, transport and equipment operators and related occupations	83 (7.1)	55 (9.5)
Natural resources, agriculture and related production occupations	18 (1.5)	5 (0.9)
Manufacturing and utilities	27 (2.3)	17 (2.9)
*Household income*		
$0 to $19,999	71 (6.1)	55 (9.5)
$20,000 to 39,999	170 (14.5)	97 (16.8)
$40,000 to $59,999	187 (15.9)	99 (17.1)
$60,000 to $79,999	174 (14.8)	88 (15.2)
$80,000 to $99,999	152 (12.9)	71 (12.3)
$100,000 to $119,999	102 (8.7)	42 (7.3)
$120,000 to $139,999	91 (7.7)	36 (6.2)
$140,000 to $169,999	70 (6.0)	27 (4.7)
$170,000 or more	69 (5.8)	24 (4.2)
Don’t know or refuse to answer	88 (7.5)	38 (6.6)
*Problem gambling status (PGSI)*		
Non-problem	604 (51.4)	169 (29.3)
Low risk	276 (23.5)	140 (24.3)
Moderate risk	185 (15.8)	161 (27.9)
Problem	109 (9.3)	107 (18.5)
Mean age	45.36 years (*SD* = 15.32)	41.94 years (*SD* = 14.83)

* Occupation was only asked for respondents who indicated they worked full-time, part-time, or casual, therefore *N* for this question = 677 for the full sample and 337 for the at risk sample.

The survey included 65 questions, although many of these were multi-item scales and question sets. Participants were advised that the survey would take approximately 20 minutes to complete.

### Measures

#### Safe gambling practices (SGPs)

The 51 practices from the formative work were condensed to 43 items by discarding five items relating to help-seeking (considered relevant only to problem gamblers), and removing three items that were similar to others from a behaviour standpoint; despite having being retained as distinct within the prior study. We operationalised the 43 items as clear statements reflecting discrete practices to which respondents could respond ‘yes’ or ‘no’ to using them within the past 12 months.

#### Outcome measures

*Short Gambling Harms Screen* (SGHS; [22]): This screen requires yes/no responses to ten gambling harm items (e.g. ‘felt like a failure’), framed as whether they were experienced as a result of one’s own gambling in the past 12 months. ‘Yes’ responses are summed. Higher scores indicate more gambling-related harm. It deliberately measures only consequential harms from gambling, and does not assess cognitions and behaviours associated with disordered gambling that are not directly harm-related. The SGHS is the only published validated instrument that exclusively measures gambling harm.

*Problem Gambling Severity Index* (PGSI; [15]): The PGSI contains nine items with four response options: ‘never’ (0), ‘sometimes’ (1), ‘most of the time’ (2), and ‘almost always’ (3). Scores are summed to categorise respondents as: non-problem gambler (0), low risk gambler (1–2), moderate risk gambler (3–7), or problem gambler (8–27). The PGSI contains items probing indicators of behavioural addiction and harmful consequences from gambling. Only four items directly relate to gambling-harm, making the PGSI conceptually distinct from the SGHS.

#### Risk factors

We reviewed the literature to identify risk factors for problematic gambling with most empirical support, along with appropriate measures. Those measured in the study were:

- *Demographic characteristics*: (see Table 1)
- *Importance of spirituality/religion*: 5-point scale (‘not at all important’ to ‘extremely important’).
- *Carer status*: whether 1) primary carer for another adult; 2) dependent on another adult for primary care
- *Early gambling experiences*: 1) age started gambling; 2) frequency of adults in household gambling when growing up; 3) frequency of gambling with or accompanying parents when they gambled; 4) whether any adults in the household had a gambling problem when growing up.
- *Frequency of gambling in the past 12 months*: 1) on nine different gambling activities; 2) alone; 3) online; 4) with a dependent; 5) with a carer.
- *Highest spend gambling activity in the past 12 months*: single question.
- *Distance from gambling venues*: 1) where participant gambles; 2) where they can play VLTs/slots.
- *Number of the friends who gamble*: single question.
- *Self-reported previous gambling problem*: 1) prior to the past year; 2) in past two years.
- *Mental disorder diagnosis from a professional*: Yes/no if ever received.
- *Currently consume tobacco products*: yes/no
- *Gambling Outcomes Expectancies Scale* (GOES; [23]): 18 items rated on a 6-point scale (‘strongly disagree’ to ‘strongly agree’). Total scores are generated for five domains of gambling motivation (social, money, excitement, escape, ego enhancement). Higher scores indicate greater strength of motivations.
- *Gambling Urge Scale* (GUS; [24]): six items rated on a 7-point scale (‘strongly disagree’ to ‘strongly agree’ to measure thoughts and feelings about gambling urges (e.g. ‘I crave a gamble right now’). Higher total scores indicate stronger urges.
- *Gambling Fallacies Measure* (GFM; [25]): ten items examining cognitive errors in gambling (e.g. ‘a positive attitude or doing good deeds increases your likelihood of winning money when gambling’). Correct responses are coded as 1. Higher total scores reflect greater resistance to gambling fallacies.
- *Brief Perceived Social Support* (BPSS; [26]): six items (e.g. ‘I receive a lot of understanding and security from others’) measured on a 5-point scale (‘does not apply at all’, to ‘exactly applicable’). Higher scores indicate greater perceived social support.
- *Kessler Psychological Distress Scale—Brief* (K6; [27]): six items pertaining to the past 30 days (e.g. ‘during the last 30 days how often did you feel nervous’), measured on a 5-point scale (‘none of the time’ to ‘all of the time’). Higher scores indicate greater psychological distress.
- *Barratt Impulsivity Scale—Brief* (BIS-B; [28]): eight items measured on a 4-point scale (‘rarely/never’ to ‘almost always/always’) measuring levels of impulsiveness (e.g. ‘I plan tasks carefully’). With some reverse-coding, higher total scores indicate greater impulsiveness.

### Statistical analysis

Our analysis aimed to evaluate the candidate SGPs in the sub-sample of gamblers who could potentially benefit from their use. This involved two stages: (1) identifying the population of gamblers most susceptible to gambling harm, and (2) evaluating the SGPs in this population. Both stages relied on a robust form of regression, ‘elastic net’.

#### Elastic net regression

In situations involving numerous potentially correlated and multicollinear predictors, ordinary least squares (OLS) regression can perform poorly in prediction and interpretation [29]. The large number of degrees of freedom, i.e. unconstrained beta coefficients, can lead to overfitting of the true effects. Interpretation is also problematic and non-intuitive, both because of the sheer number of free parameters, and also due to a phenomenon whereby beta estimates are highly interdependent. The estimated value of one beta can depend largely on the estimates of other beta coefficients, meaning that beta values can change substantially if one or more predictors are excluded from the model. This is inconsistent with the natural and desired interpretation of regression coefficients as a set of distinct and largely independent effects.

Classically, large candidate sets of predictors have been handled via different algorithms for selecting a smaller subset of predictors, including stepwise variable selection techniques. However, these methods are extremely sensitive to the peculiarities of any one dataset because of the inherent discreteness and tendency to find local optima [30]. A stable and robust alternative is to introduce an additional penalty term to the standard OLS criterion, which is to minimise the sum of squared errors (SSE). Ridge regression minimises not only SSE, but also the *L*_2_ norm (i.e. sum of squares) of the beta coefficients themselves [31]. Similarly, the ‘lasso’ [32] penalises the *L*_1_ norm, which is the summed absolute value of the coefficients. Whilst ridge regression tends to penalise overly large beta coefficients, the lasso tends to drive less useful coefficients to zero–essentially performing variable selection. Both methods encourage ‘efficiency’ in beta coefficients, as the estimator balances the dual criteria of maximising both predictive performance and model parsimony.

The elastic net method (R package *elasticnet*) incorporates advantages of both ridge regression and the lasso, incorporating both *L*_1_ and *L*_2_ norms in the penalty term [29]. Two meta-parameters determine the amount of penalisation, and the *L*_1_ versus *L*_2_ balance, which are estimated via cross-validation. The practical advantages over OLS regression in this context are: 1) overfitting is largely prevented as model complexity is intrinsically constrained by ability to generalise; 2) many potential candidate predictors can be considered; 3) beta coefficients tend to reflect uncorrelated and unique effects, improving interpretation; and 4) less useful coefficients are driven to zero, yielding a robust form of variable selection. All these features are useful in both stages of analysis. Given elastic net regression is a robust procedure that automatically handles multicollinearity, and given all binary predictors (i.e. use of SGPS) and reasonably large sample size, the method only assumes that the response is a continuous variable.

#### Identifying the population

Elastic net regression was used to create an operational definition of gamblers most susceptible to experiencing gambling-related harm. The predictor variable set comprised all risk factors for gambling-related harm, and the outcome was SGHS score [22]. The predicted scores of this model represent a measure of vulnerability in that they reflect the expected value of harm, integrating information from all available risk factors. We defined this population as those having an expected value of 1+ harms, regardless of whether or not they had actual reported harms. These gamblers do not necessarily experience harm but still experience risk; consequently, this group also included some unharmed gamblers. The elastic net allowed us to incorporate a large number of correlated risk factors in making the estimation of an expected value of 1+ harms, whilst preventing overfitting to the data.

#### Evaluating the SGPs

The second analysis step was to evaluate the SGPs with respect to the restricted set of gamblers most susceptible to experiencing gambling-related harm. We excluded gamblers who were not susceptible to harm from this analysis, since some people may not use SGPs simply because they do not need to. Elastic net regression was also employed in evaluating the SGPs, using all candidate SGPs as predictors, and the SGHS score again as the predicted outcome amongst the subset of vulnerable gamblers. Multivariate regression is intrinsically geared towards identifying predictors with unique explanatory power. However, as described above, the elastic net variant provides an additional advantage in that it accomplishes implicit variable selection. That is, it identifies the smallest set of SGPs that are instrumental in affecting the outcome. Negative parameters indicate that use of a SGP is associated with a reduction in harms; positive coefficients with an increase in harms.

### Ethics

The study procedures were carried out in accordance with the Declaration of Helsinki. The Institutional Review Board of the University of Alberta approved the study. All subjects were informed about the study via a participant information sheet that detailed the study’s aims, investigators, survey topics, survey length, voluntary participation, that they could withdraw at any time, that the survey was anonymous, the security of data storage, publication of aggregated results, ethics approval number and contact details of the approving ethics office. All participants provided informed consent by clicking ‘yes’ to confirm that they were 18 years or over and ‘yes’ to confirm that they were providing informed consent to participate in the study.

## Results

Table 2 presents the standardised elastic net regression coefficients for the risk model predicting harm from all risk factors as the first step of the analysis: identifying the restricted set of gamblers most susceptible to experiencing gambling-related harm. Note that standard errors / p-values usually associated with regression models are not applicable to elastic nets. However, the coefficients themselves indicate relative variable importance, in the context of all other predictors. The risk model explained 40.1% of the variance in harm scores. The most important predictor of (expected) current gambling harm was the existence of gambling problems prior to the past two years (*b* = .77), followed by the presence of gambling urges (.63), impulsivity (.27), and when as a child, adults in the household had a gambling problem (.26).

**Table 2:** Standardised elastic net regression coefficients predicting harm from all risk factors.

Variable	Coefficient
(Intercept)	1.53
Gambling problems prior to the past 2 years	0.77
*Highest gambling spend*	
VLTs / slots (reference group)	-
Instant win tickets	-0.13
Sports betting	0.00
Horse race betting	0.00
Keno	0.00
Bingo	0.04
Casino table games	0.00
ESports	0.00
Fantasy sports	-0.06
If gambled online	0.18
Friends who gamble regularly	0.00
Perceived social support (BPSS)	-0.13
*Gambling Outcomes Expectancies Subscales*	
Excitement	0.02
Escape	0.00
Ego	0.00
Money	0.15
Social	0.00
Gambling Fallacies (GFM)	-0.09
Gambling Urges (GUS)	0.63
Age	0.00
Canadian born	0.00
*Residence*	
Calgary (reference group)	-
Edmonton	0.00
Regional town	0.00
Small town	0.00
Rural or remote location	0.00
Gender	0.00
*Language spoken at home*	
English (reference group)	-
French	0.00
Other	0.00
*Indigenous status*	
Non-Aboriginal (reference group)	-
First Nation	0.04
Métis	0.00
*Marital status*	
Single/never married (reference group)	-
Living with partner/defacto	0.00
Married	0.00
Divorced or separated	0.00
Widowed	0.00
*Living arrangements*	
Live alone (reference group)	-
Couple (no dependents)	0.00
Couple with at least one dependent child	-0.12
Couple living with independent child(ren)	0.00
Single parent living with at least one dependent child	0.00
Single parent living with independent child(ren)	0.00
Share house with other adults	0.00
Live with parents	0.02
Other	0.00
Education	0.00
*Work status*	
Work full-time (reference group)	-
Work part-time or casual	0.00
Self-employed	0.00
Unemployed and looking for work	0.05
Full-time student	0.00
Full-time home duties	0.00
Retired	0.00
Sick or disability pension	0.00
Other	0.00
*Occupation*	
Business, finance and administration (reference group)	-
Management	0.00
Natural and applied sciences and related occupations	0.00
Health	-0.01
Education, law and social, community and government services	0.00
Art, culture, recreation and sport	0.00
Sales and service	0.00
Trades, transport and equipment operators and related occupations	0.00
Natural resources, agriculture and related production occupations	0.00
Manufacturing and utilities	0.00
NA	0.00
Income	-0.01
Disposable income	0.00
Primary carer for another adult	0.00
Dependent on another adult for care	0.00
Importance of religion	0.01
When a child, other adults gambling	0.00
When a child, gambled with adults	0.00
When a child, adults had gambling problems	0.23
Distance from VLT venue	0.00
Distance from gambling venue	-0.06
Age started gambling	0.01
Mental disorder diagnosis	0.09
Impulsivity (BIS)	0.27

The expected number of harms for each respondent, given knowledge of their risk factors, was generated from the risk model. Of the 1,174 cases analysed, 577 cases had an expected harms score equal to or greater than one. For the second step in the analysis, a second elastic net again predicted harm scores for these 577 gamblers, using the SGPs as candidate predictors. Table 3 provides the SGPs associated with higher or lower degrees of harm amongst these gamblers. Negative coefficients indicate SGPs that are associated with less gambling harm; positive coefficients indicate those that are associated with more gambling harm. The behaviour most strongly associated with increased harm (*b* = 2.08) was using credit card cash advances to gamble. The behaviour most strongly associated with reduced harm was ‘If I’m not having fun gambling, I stop’ (*b* = -1.07).

**Table 3:** Standardised elastic net regression coefficients predicting harm from use of SGPs (*N* = 577).

**Most effective SGPs**	**Coefficient**
1. If I’m not having fun gambling, I stop	-1.07
2. I keep a household budget	-0.64
3. I have a dedicated budget to spend on gambling	-0.52
4. My leisure time is busy with other hobbies, social activities and/or sports	-0.51
5. If I’m feeling depressed or upset, I don’t gamble	-0.33
6. When I gamble, I always set aside a fixed amount to spend	-0.25
7. I research systems or strategies for success at gambling	0.50
8. I use gambling to make money / supplement my income	0.60
9. I have used cash advances on my credit card to gamble	2.08
**Remaining SGPs**	**Coefficient**
When I make a large win at gambling, it is time for me to quit	-0.15
I only use gambling winnings for fun activities or purchases	-0.11
I don’t use gambling winnings to pay bills	-0.11
As a rule, I don’t go gambling just to avoid being bored	-0.11
I don t gamble when I have consumed alcohol or drugs	-0.04
I make sure I take regular breaks (at 30min, 1 hour, etc.) when gambling	-0.01
I restrict myself to gambling only on one or two days a week, or less often	0.00
I restrict myself to gambling only in the evenings	0.00
I have a rule that I only gamble for an hour (or 1/2 hour, etc.) at a time	0.00
I always gamble for a fixed amount per spin/bet/etc.	0.00
I only gamble on my favourite team, game or event	0.00
If I’m losing after an hour (or 1/2 hour, 2 hours, etc.) of gambling, my rule is to quit	0.00
I keep a record of how much I spend on gambling	0.00
I study the gambling odds before I play	0.00
Before I gamble, I make a point to think about how long it took me to save the money	0.00
I always read the fine print on gambling promotions before I participate	0.00
I don t gamble just because my friends are gambling	0.00
I won’t go out with friends if I think that they will encourage me to gamble	0.00
I don t gamble with friends who like higher stakes than I do	0.00
When I feel myself getting too emotional about gambling, I take a break	0.00
I have set up a spending limit on my gambling membership or loyalty account(s)	0.00
I only gamble with the one betting account	0.00
I deliberately ignore or don’t read gambling advertisements or promotions	0.01
Before I gamble, I make a point to think about how I will feel if I lose the money	0.01
I practice my skills at gambling	0.05
I don t allow myself to look at gambling websites at work	0.13
I choose my online betting website(s) because they offer daily spend limits	0.16
I always leave my bank cards at home when I gamble at venues	0.16
I make a point of thinking about my family when I gamble	0.17
I have set up a deposit limit(s) on my online betting account(s)	0.22
Before I gamble, I make a point to think about what else I could do with the money	0.24
I have a rule that I don’t go gambling alone	0.25
As a rule I don’t gamble in the company of an adult who I am the primary carer for, or who is my primary carer	0.37
I often talk about gambling with my friends and/or family	0.46

The top portion of Table 3 identifies the most effective SGPs, addressing the first aim of the study. Our primary criteria for selection was efficacy in independently predicting gambling related harm. However, importantly, two SGPS with strong effect sizes were manually excluded (last two rows in Table 3) from the list of effective practices, based on item content. We screened the top performing SGPs based on whether they could be framed as positive, general advice to gamblers. For example, the positive coefficient for ‘I often talk about gambling with my friends and/or family’ suggests that this is an indicator of a problematic preoccupation with gambling. However, it would be clearly unhelpful to advise gamblers not to discuss their gambling with their family. The other excluded item assumes that the respondent is in a primary carer relationship, and therefore is unsuitable as general advice.

Of the selected items, 1–6 were most strongly associated with reduced gambling harm and therefore also represent the most likely efficacious practices to use for gambling to be non-harmful. Items 7–9 were most strongly associated with increased gambling harm, and represent the most evident practices to avoid. Our cut-off point of nine SGPs is somewhat arbitrary, although it was also a choice-point informed by their appropriateness for consumer messaging and guidelines. An expanded set of effective SGPs might be useful for other purposes.

To address the study’s second aim, we examined how widely the nine most evidently important SGPs were used by gamblers who are most susceptible to experiencing gambling-related harm (Table 4).

**Table 4:** Frequency of use for the most important SGPs amongst gamblers (*N* = 577).

SGP	*n*	%
*Associated with reduced harm*:		
1. If I’m not having fun gambling, I stop	469	81.3
2. I keep a household budget	415	71.9
3. I have a dedicated budget to spend on gambling	261	45.2
4. My leisure time is busy with other hobbies, social activities and/or sports	429	74.4
5. If I’m feeling depressed or upset, I don’t gamble	258	44.7
6. When I gamble, I always set aside a fixed amount to spend	412	71.4
*Associated with increased harm*:		
7. I research systems or strategies for success at gambling	147	25.5
8. I use gambling to make money / supplement my income	199	34.5
9. I have used cash advances on my credit card to gamble	139	24.1

Each SGP associated with reduced gambling harm was used by over 70% of these gamblers, except for ‘I have a dedicated budget to spend on gambling’ and ‘If I’m feeling depressed or upset, I don’t gamble’, each used by only 45% of this group. Approximately three-quarters of these vulnerable gamblers reported *not* using each SGP associated with increased gambling harm, except for ‘I use gambling to make money / supplement my income’ which 65.5% did not use.

The third aim was to assess whether use of SGPs differs by gambler characteristics. A total SGP score was calculated by scoring +1 for use of SGPs items 1–6 and -1 for items 7–9. Non-parametric tests examined relationships between total SGP score and the independent person-variable predictors. A Mann-Whitney U test examined gender and SGP scores. Spearman’s correlations were examined between total SGP score and (in-turn): age, PGSI score, K6 score and BIS score. Kruskall-Wallis tests examined differences in SGP scores and (in-turn): PGSI group, gambling frequency, and highest spend gambling activity. Where significant differences were found, Mann-Whitney U tests, with Bonferroni corrections, were performed as post-hoc analyses.

Males (*m* = 2.81) used significantly fewer SGPs compared to females (*m* = 3.23); *U* = 34424.50, *z* = -2.71, *p* = 0.01, *r* = -0.11; but SGP score was not correlated with age. Those using more SGPs had lower psychological distress, *r*_s_ = -0.19, *p* < 0.001, and impulsivity, *r*_s_ = -0.24, *p* < 0.001.

Respondents using more SGPs had lower PGSI scores, *r*_s_ = -0.49, *p* < 0.001, and significant differences were found between PGSI groups; *H*(3) = 150.51, *p* < .001. Non-problem gamblers (*m* = 3.91) had significantly higher SGP scores than moderate risk (*m* = 2.75; *r* = -0.35) and problem gamblers (*m* = 1.18; *r* = -0.63). Low-risk gamblers (*m* = 3.78) had significantly higher SGP scores than moderate risk (*r* = -0.31) and problem gamblers (*r* = -0.62). Moderate risk gamblers had significantly higher SGP scores than problem gamblers (*r* = -0.40).

Significant differences were found between gambling frequency and SGP scores; *H*(4) = 24.38, *p* < .001. Those gambling once a month (*m* = 3.59) used more SGPs than those gambling 2–3 times a week (*m* = 2.88; *r* = -0.18) and 4+ times a week (*m* = 2.16; *r* = -0.31). Those gambling 2–3 times a month (*m* = 3.01) used more SGPs than participants who gambled 4+ times a week (*r* = -0.20).

We examined relationships between SGP score and highest spend gambling activity. Some activities were excluded due to their low prevalence, including: bingo (*n* = 34), eSports (8), fantasy sports (9), horse racing (11), keno (3), and sports betting (30). Significant differences were found between SGP scores and the three activities used in the following analysis (instant win tickets (*n = 192*), VLTs/slots (206), and casino table games (84)); *H*(2) = 13.73, *p* = .001. Respondents who spent the most money on instant win tickets (*m* = 3.42) used more SGPs than those whose highest spend activity was VLTs/slots (*m* = 2.71; *r* = -0.18).

## Discussion

Previous research has identified self-regulatory strategies used more by lower-risk than higher-risk gamblers [16, 18–19]. Our study extends this research by identifying a set of nine safe gambling practices that best prevent gambling-related harm amongst those most susceptible to experiencing harmful consequences from their gambling. It is important to note that these nine SGPs are not the only practices that can help to protect against harmful gambling, and that other practices promoted on gambling-related websites, in player information, in the broader media and by treatment professionals can also be useful. The nine SGPs are those that were the most protective amongst the much larger group of change strategies that gamblers can use to self-regulate their gambling, and therefore can provide the basis of an evidence-based set of guidelines.

Our parsimonious set of nine SGPs can be expressed as the following safe gambling guidelines:

- If you’re not having fun gambling, stop.
- Keep a household budget.
- If you gamble, have a dedicated budget for your gambling.
- Engage in other leisure activities, hobbies, social activities or sports.
- Do not gamble if you’re feeling depressed or upset.
- When you gamble, always set aside a fixed amount you can spend.
- Do not use credit, or cash advances on your credit card, to gamble.
- Do not use gambling to make money or supplement your income.
- Do not think that systems or strategies will ensure your success at gambling.

While the nine SGPs may sound simplistic, their effective implementation requires gamblers to enact several broader cognitive-behavioural change strategies that can be used to self-regulate gambling [20]. These include strategies relating to limiting finances (keep a household budget), controlling consumption (having a dedicated gambling budget, setting aside a fixed amount to spend, not using credit to gamble), avoidance of certain behaviours (not gambling if depressed or upset, stop gambling if not having fun), behaviour substitution (engage in other leisure activities), and cognitive strategies (not thinking systems or strategies will help you win, not using gambling to make money). The strategies relate both specifically to gambling (e.g., not gambling when upset or depressed) and to practices that are not specific to gambling (e.g., keep a household budget, engage in other leisure activities), so their effective implementation requires changes beyond gambling behaviour alone. Importantly, the nine SGPs encompass both distal (pre-gambling) and proximal (during gambling) strategies [33]. Distal strategies include, for example, keeping a household budget, setting a dedicated budget for gambling, allocating a fixed amount that one can gamble before commencing gambling, and engaging in other leisure activities. Proximal strategies, such as stopping gambling if not having fun, require gamblers to take actions during a gambling session, which is likely to be more difficult than adhering to distal strategies. Many gamblers find it difficult to limit their gambling during play when they may feel excited, frustrated, emotional, dissociated, vulnerable to erroneous beliefs, subject to peer pressure, and tempted to chase losses [34–35], and effective strategies to manage gambling urges appear to be particularly challenging [20].

Effectively implementing behaviour change strategies, such as the nine SGPs, requires adequate action and coping planning between intentions and behaviour in order to realise the behavioural goal [36–37]. The process requires pre-decisional strategies to form intentions to achieve a desired goal (e.g., reducing or quitting gambling), pre-actional strategies by using planning to initiate the intention or goal, actional strategies to implement the behaviour, and post-actional strategies to evaluate it outcomes [38]. Coping planning is also needed to identify situations and barriers where goals may be undermined [38]. Additional research is needed to understand how action and coping planning can best support the implementation of the nine SGPs.

Currently, each of the nine SGPs is promoted on various gambling help, government and gambling industry websites, but these websites often do not include all of these SGPs and often instead include practices with lower demonstrated efficacy in protecting against gambling-related harm. In contrast, the guidelines developed in this study could be consistently promoted on gambling-related websites and apps, in public health materials, and in gambling venues. Market testing might optimise wording to ensure resonance and comprehension. These guidelines can also form a consumer self-assessment test, ideally with automated personalised feedback that identifies practices to make an individual’s gambling safer. Gambling treatment providers might also use the guidelines to provide practical advice to clients on cognitive-behavioural change.

The use of SGPs can also be measured at a population level. Prevalence studies rely on problem gambling screens to track changes in maladaptive gambling behaviour. However, the prevalence of problem gambling is too low to reliably detect changes between assessment periods. Instead, prevalence studies could measure the use of SGPs to detect changes in safe gambling behaviour, which would be more reliable, given the much greater prevalence of SGP use in a population. Such assessments would be particularly useful to evaluate the efficacy of new harm minimisation initiatives, as well as changes in policy and practice that might be expected to impact on harmful gambling.

This study can inform harm minimisation efforts in Alberta, as well as across wider locations. Certain evidently helpful SGPs were practised by only a minority of gamblers who are susceptible to experiencing gambling-related harm; specifically having a dedicated budget to spend on gambling, and not gambling when feeling depressed or upset. Public health messaging promoting these practices may help to increase their uptake. Male gamblers, more frequent gamblers especially on VLTs/slots, and gamblers with higher impulsivity, psychological distress and PGSI scores are most likely to experience gambling-related harm, but are less likely to use SGPs. This knowledge can inform public health communications which can be tailored accordingly in terms of target audiences, appropriate messages, and use of relevant media.

Given that public health messaging is rarely sufficient on its own to change behaviour [39], the SGPs should also be used to change policy and practice. In addition to promoting the guidelines, gambling regulators and operators could facilitate use of the SGPs. They could provide budgeting tools to encourage gamblers to calculate an affordable gambling budget in the context of their overall household budget. Operators could provide pre-commitment systems to facilitate limit-setting prior to gambling. They could avoid extending credit for gambling and prevent customers using credit cards to gamble. Operators and regulators should ensure that gambling advertising does not encourage faulty cognitions, such as suggesting that certain systems or strategies will enhance the chances of winning. Identifying these specific changes to policy and practice that directly relate to the SGPs does not preclude the need for additional reforms to overcome the limitations of responsible gambling [4–5, 40–41], but discussion of these reforms is beyond the scope of the current paper.

### Limitations and future research

Data were collected only in Alberta with a modest sample size. While reasonably balanced by gender, age and other demographic characteristics, our convenience sample was unlikely to be representative of the population of gamblers. Replicating the study in other locations and with larger and more representative samples is needed to confirm the results. Some variation in the uptake of SGPs may be expected in different locations, given that socio-economic characteristics, cultural norms, legal gambling forms, their accessibility and marketing vary. Further, some comprehensive studies of self-regulatory practices used by gamblers have been published since the survey was conducted for the current research, and should be considered in future research to inform the set of SGPs tested [20–21, 33]. As noted earlier, the safe gambling guidelines would benefit from market testing to optimise wording to ensure resonance and comprehension. For example, ‘ …have a dedicated budget for your gambling’ might be clearer as ‘ …have a dedicated budget for your gambling and stick to it’. As noted earlier, research is needed to understand how action and coping planning can best support the implementation of the nine SGPs. Finally, evaluation studies could examine the efficacy of the guidelines across different forms of gambling, and over time in longitudinal designs.

## Conclusion

To our knowledge, this study has developed the first evidence-based set of safe gambling practices whose use predicts the absence of gambling-related harm amongst gamblers who might otherwise be expected to experience harm. As safe gambling guidelines, they provide practical direction for consumers on how to avoid harmful gambling behaviours and consequences. They can be further used to measure the prevalence of safe gambling and changes over time at the population level, and to inform supportive changes in gambling policy and practice.
