# Supplementary material for: Avoiding gambling harm: An evidence-based set of safe gambling practices for consumers
Source: PLoS One. 2019 Oct 17;14(10):e0224083. doi: 10.1371/journal.pone.0224083 (PMC6797237; doi:10.1371/journal.pone.0224083)
Supplement: Attachment — Submitted filename: Response to Reviewers.docx [file pone.0224083.s001.docx]

Journal Requirements:

1. When submitting your revision, we need you to address these additional requirements.

Please ensure that your manuscript meets PLOS ONE's style requirements, including those for file naming. The PLOS ONE style templates can be found at
[http://www.journals.plos.org/plosone/s/file?id=wjVg/PLOSOne_formatting_sample_main_body.pdf](https://protect-au.mimecast.com/s/STtrCMwvYnhRVYwMtJuw8o?domain=journals.plos.org) and [http://www.journals.plos.org/plosone/s/file?id=ba62/PLOSOne_formatting_sample_title_authors_affiliations.pdf](https://protect-au.mimecast.com/s/2GTBCNLwZoiE5pODcRl75P?domain=journals.plos.org)

Response: We have reviewed these requirements and ensure the manuscripts conforms to this

2. Please provide additional details regarding participant consent. In the ethics statement in the Methods and online submission information, please ensure that you have specified (1) whether consent was informed and (2) what type you obtained (for instance, written or verbal, and if verbal, how it was documented and witnessed).

Response: We have now included this information in the Methods section and the online submission information

3. We note that you have indicated that data from this study are available upon request. PLOS only allows data to be available upon request if there are legal or ethical restrictions on sharing data publicly. For information on unacceptable data access restrictions, please see [http://journals.plos.org/plosone/s/data-availability#loc-unacceptable-data-access-restrictions](https://protect-au.mimecast.com/s/KFV1COMx1ph0rk7zcPBLN9?domain=journals.plos.org).

In your revised cover letter, please address the following prompts:

a) If there are ethical or legal restrictions on sharing a de-identified data set, please explain them in detail (e.g., data contain potentially identifying or sensitive patient information) and who has imposed them (e.g., an ethics committee). Please also provide contact information for a data access committee, ethics committee, or other institutional body to which data requests may be sent.

b) If there are no restrictions, please upload the minimal anonymized data set necessary to replicate your study findings as either Supporting Information files or to a stable, public repository and provide us with the relevant URLs, DOIs, or accession numbers. Please see [http://www.bmj.com/content/340/bmj.c181.long](https://protect-au.mimecast.com/s/qSM7CP7y2qcoAnj8crRsCe?domain=bmj.com) for guidelines on how to de-identify and prepare clinical data for publication. For a list of acceptable repositories, please see [http://journals.plos.org/plosone/s/data-availability#loc-recommended-repositories](https://protect-au.mimecast.com/s/H8qeCQnz3rU34L2Mu9q_n1?domain=journals.plos.org).

We will update your Data Availability statement on your behalf to reflect the information you provide.

Response: We have addressed this in our revised cover letter and the online submission form

**Reviewer #1: PONE-D-19-19226**
Avoiding gambling harm: An evidence-based set of safe gambling practices for consumers

Thank you for the opportunity to review the above manuscript. The manuscript presents results of an online survey which identified gambling behaviour practices predicting a reduction of harm or increase in gambling harm. The result is a proposed set of 9 evidence-based gambling practices indicated to support safe gambling for consumers.

There are a number of strengths to the current study, including addressing an important gap in the current literature for evidence-based support of gamblers in reducing gambling behaviour, the considered analytic techniques used and the use of a specific, targeted sample. Overall, the paper was well written and presented, clear aims that were met with appropriate methodology and analysis, and interpretation of how results could be utilised in the applied setting.

Response: Thank you for your positive comments

I have a number of comments and queries for the authors to consider. Given the limitations of the market sample recruitment (to be detailed), lack of representativeness suggested, and the somewhat arbitrary selection of SPGs, I suggest that there are particular aspects that need to be addressed prior to wide dissemination and application of this particular set of 9 SPGs. In particular, more details and/or justification regarding the selection of the 9 SPGs based on the results and the imbalance between the 9 that reduce (practices to do n=6) and those that increase (practices to not do, n=3).

Abstract
Strong summary of paper, albeit not including the 9 SPGs.

Response: We have now summarised the 9 SGPs in the Abstract.

Introduction
Good introduction to paper. Important to note that strategies are being recommended, advertised and used by professionals and consumers alike, with limited or no evidence of effectiveness. The aim is therefore an important one to address.

Response: Thank you for your positive comments.

Method
- Additional details required to explain the approach taken for identifying potential SGPs; the literature review (e.g., search terms, scope) and content analysis (e.g., consumer practices and/or professional recommendations); actual use vs recommended use, etc. The authors indicate that details are reported elsewhere (deidentified for submission purposes), making it difficult to review these processes here.

Response: We have now expanded this section to include the details requested, as well as added the formerly deidentified reference.

- How did the research/treatment/training/policy professional group determine helpfulness of the each identified practice? Professional judgement or other evidence-based, local, unpublished, grey literature data perhaps? Could these professionals add additional practices, or was there an indication that the list was exhaustive? Rating scale and cut-off for low, resulting in discarded practices, not detailed, nor how differences between group decided.

Response: We have now expanded this section to include the details requested.

- Participants were to have engaged in ‘at least monthly gambling’ – over what period of time? Presuming 12 months, given that the response to practices was whether used within past 12 months, but not clear?

Response: We have clarified this was over the past 12 months.

- Total number of items in survey and estimated duration for completion

Response: We have now included the number of items in survey and estimated duration for completion.

- Proportion of people commencing and completing survey – missing data?

Response: In the ‘participants and procedure’ section we have now included the starting sample size and specified the reasons and number of excluded participants.

Results
- I was not familiar with the specific form of regression used, elastic net regression prior to reviewing this paper, and therefore appreciated the details provided. Were there specific assumptions that were tested and met prior to analysis?

Elastic net regression has very weak assumptions in the present context, it being designed to be robust to multicollinearity, and our sample size being reasonably large (normality of residuals not strictly required), and predictors are all binary (linearity with respect to predictors not applicable). This is now explained at the end of the analysis section.

- The selection of the final 9 SGPs requires some more clarification. Although as indicated by the authors, selection was somewhat arbitrary (line 254), I am not clear as to why the emphasis is on reducing gambling a(6 items) compared to increased gambling harm (3 items). Also, why stronger performing SGPs were not included. For example, “I often talk about gambling with my friends and/or family” (0.46) and “As a rule, I don’t gamble in the company of an adult who I am the primary carer for, or who is my primary carer (0.36) are stronger than SGPs 5 and 6 which are included. I would be interested in seeing perhaps the top 5 performing for reducing gambling and the top 5 performing for increased gambling harm, and then those selected to form the “most evidently important SGPs”. I suggest that understanding this selection process more fully is required. I appreciate that some SPGs were selected as more readily translatable, however by excluding a focus on some items, those working with individuals may miss important details (such as those with an adult carer, for example). Presenting the top performing SPGs is a missed step, particularly given the emphasis on the evidence-based approach to identifying strategies.

Our apologies: in the submitted draft we neglected to explain the rationale for manual rejection of two higher-performing items, based on item content and our criteria that selected items could be framed as positive, general advice for gamblers. We have inserted a paragraph after Table 3 that explains our screening approach, and rationale for excluding these two items.

- Potential limitations as distinctions between, for example, Having a budget for gambling vs sticking to the budget for gambling. How was this considered?

Response: We are not entirely clear what the reviewer is asking us to do here. However, we have added in the limitations and future research section that “the safe gambling guidelines would benefit from market testing to optimise wording to ensure resonance and comprehension. For example, ‘...have a dedicated budget for your gambling’ might be clearer as ‘...have a dedicated budget for your gambling and stick to it’.

- SPG 8 – 34.5% while text reports 65% (line 268-269) – should read ‘did not use’ (as done in first phrase of sentence, not latter)

Response: We have now made this change.

- Table 4 heading to include N or sub-set of gamblers in title

Response: We have now added N = 577 to this table heading

- Table 6 – for consistency, don’t to be do not

Response: We have now made this change.

Discussion
- Well considered potential application of the SPGs broadly, however, as with other work examining the use of strategies, this study does not indicate how gamblers should implement such recommended strategies. Aspects of when, how, with who, etc are not detailed, making appropriate application of the strategy more difficult perhaps. Reference to mechanisms such as implementation intentions, action and coping planning etc. could support those wishing to action such SPGs.

Response: We have now included a discussion of implementation intentions, action and coping planning for effective implementation of the nine SGPs, though note that these were not directly considered in our study but are a useful focus of further research.

- Did the authors wish to comment on the strategies that were not predicting increase/reduction of harm that they would have anticipated would have been relevant or are reported as popularly used but not predictive; particularly given all items included had been indicated as helpful by the professional group?

Response: We have decided not to comment on the other SGPs. This is because our nine SGPs represent those that best prevent gambling-related harm amongst those most susceptible to experiencing harmful consequences from their gambling. These nine SGPs are not the only practices that can help to protect against harmful gambling, and other practices promoted on gambling-related websites, in player information, in the broader media and by treatment professionals can also be useful. We have now added this point early in the discussion section.

- Were strategies/practices identified in more recent work, included in the initial pool of practices (unable to determine from manuscript details), or have some potentially effective practices recently identified (e.g., in work by Rodda and colleagues) been missed due to timing and could be considered in future? How do the effective practices sit within the different types/groups of change strategies identified? For example, I would also be interested in a note regarding the timing of the SPGs; that is, those that are enacted pre-gambling, and others while gambling, or those specific to gambling and not (e.g., keeping a household budget).
o Rodda, S. N., Bagot, K.L., Cheetham, A., Hodgins, D. C., Hing, N., & Lubman, D. I. (2018). Types of change strategies for limiting or reducing gambling behaviours and their perceived helpfulness: A factor analysis. Psychology of Addictive Behaviors, 32(6), 679-688.
o Rodda, S.N., Bagot, K.L., Manning, V., & Lubman D.I. (2019). ‘Only take the money you want to lose’ strategies for sticking to limits in electronics venues, International Gambling Studies, pp1-19. Published on line 24 May 2019.
o Rodda, S.N., Bagot, K.L., Manning, V., & Lubman D.I. (2019). “It was terrible. I didn’t set a limit”. Proximal and distal prevention strategies for reducing the risk of a bust in gambling venues. Journal of Gambling Studies, pp1-15. Published on line, 29 January 2019.

Response: The survey for the study was conducted in Nov-Dec 2017, prior to the publication of the articles cited above. We have now included reference to these recent papers in our limitations section to promote their consideration in future studies. We have also included reference to them in identifying how the SGPs sit within the different types of change strategies, whether they are distal or proximal, and whether they relate to gambling or not.

- As the initial sources of practices were both academic (literature review) and presumably applied (web site content), a note as to the source of the effective practices may be warranted. This inclusion would allow an indication as to whether web sources are an appropriate avenue for gamblers to locate effective strategies. As it is likely that individuals seeking to change their behaviour would be able to readily access on-line sources, this is an important note to include.

Response: The content analysis of the websites was conducted several years ago (2015-16), and their content is likely to have changed and will continue to change. Instead we have included the following note: “Each of the nine SGPs is promoted on various gambling help, government and gambling industry websites, but these websites often do not include all of these SGPs and often instead include practices with lower demonstrated efficacy in protecting against gambling-related harm. In contrast, the guidelines developed in this study could be consistently promoted on gambling-related websites and apps, in public health materials, and in gambling venues.”

- Finally, given the heteregenous group of gambling behaviours of the selected sample, how these practices may or may not apply across different types of gambling may be considered by future evaluation.

Response: We have now included this as a suggestion for further research.

Congratulations to the authors on this work which I expect to be of great interest to those working and researching in this area.

Response: Thank you

**Reviewer #2**

This manuscript reports on the development of a 9-item guideline of safe gambling practices (SGPs) which can be used for reducing gambling harms among gamblers and also for estimating prevalence of safe gambling on a population level for public health purposes. This manuscript, extends the existing body of knowledge by looking into the strategies’ relationship to gambling harms and by making focus on proactive strategies. The study had three aims: 1) to identify SGPs that best predict non-harmful gambling; 2) examine their frequency; 3) examine the relationship between SPGs use and gambler characteristics. The study recruited a convenience sample of 1,174 gamblers. Analysis utilised elastic net regressions to increase predictability of the models. The authors reported nine SGPs which were most effective for reduction of gambling harms. The dual use of the guidelines – as an intervention for gambling harm reduction and as a public health tool for informing gambling policy, - is particularly interesting. Overall, the manuscript is of interest for health professionals and researchers in the gambling field. Some minor corrections may improve the readability.

Response: Thank you for your positive comments.

Abstract and introduction

The abstract provides good summary of the study. However, the meaning of the opening sentence can be clarified as it is not clear how ‘infrequent’ use relates to the strategies being ‘protective’ (line 25-26).

Response: We have now clarified this sentence.

Introduction explains the background of this study well and provides good justification for it. However, a wider use of literature would make the argument stronger. The whole introduction is based on only 9 publications. In-text citations can be more precise as not all statements are supported by a reference (e.g., lines 51 and 54) which makes it difficult for a reader to check the manuscript’s claims.

Response: We have now included a wider selection of literature to better set the context for the study.

The rational of the study is clear, but summarising it at the end of the introduction before the aims would make for a good presentation.

Response: We have now summarised the study’s rationale immediately before stating its aims.

Figures and tables

There are six tables in this manuscript. The numbering of the tables should follow the order of appearing in text. However, Table 4 is referenced straight after Table 1 (line 124). Table 2 and Table 6 do not appear to be tables to me. Consider presenting the items from these tables as bulleted text, not a table. Table 3 formatting is not consistent, e.g., ‘Income’ needs to be in italics and sub-items need to be indented. Also consider merging Tables 4 and 5 and presenting frequencies for the ‘remaining SGPs’.

Response: We have now removed the early reference to Table 4 to ensure all tables are referred to and presented sequentially. We have converted Tables 2 and 6 into bullet points. In table 3 (now Table 2), we have left the formatting as is, as income and all items below it are single measures and not sub-items of a measure.

Methods

The manuscript presents a sound well-designed study appropriate for meeting the aims of this research. The limitations of the modest sample size are discussed in ‘Limitations’ and are not of a concern.

Response: Thank you for your positive comments.

Please consider reporting the study period more precisely, e.g., month (line 108). Also consider explaining the incentives to the participants a bit more (line 109) for replication purposes.

Response: We have now provided this additional information.

Results, discussion, conclusions
Results of the study support the conclusions drawn by the authors. The results contribute to a better understanding of behavioural strategies used for gambling harm reduction and provide empirical evidence of their effectiveness.

Response: Thank you for your positive comments.

Line 269: The proportion of people who used this strategy is indicated 65% while Table 5 reports 34.5%. My understanding is that the confusion comes from the wording of this sentence.

Response: This wording has now been clarified.

Line 279 and onwards in ‘Results’ section: Please check what symbol to use for reporting sample means. The manuscript uses capitalised M. Also consider reporting statistics for means, e.g., standard deviations or confidence intervals.

We have replaced M with lower case m, to be in keeping with convention that this indicates a sample, rather than a population mean. We suggest that including standard errors or CIs would decrease readability and not contribute much to these descriptions, over and above the statistical comparisons, as error bounds for these mean estimates are not core to the goals of the paper.

Lines 295-301: The manuscript reports frequencies of gambling activities of low prevalence only, while the reader may be also interested in the frequencies of more prevalent gambling activities in relation to SGP score.

Response: We have now added the frequencies in the manuscript (see paragraph just before the Discussion section)

Line 328: This sentence creates an impression that the findings of this study will be applicable only in Alberta. It appears to me that these findings can inform harm minimisation efforts across wider locations.

Response: We have now changed to read “This study can inform harm minimisation efforts in Alberta, as well as across wider locations.”

Lines 332-334: Please consider rephrasing this sentence for better clarity.

Response: We have now rephrased this sentence for clarity.

Line 347: Abbreviation RG was not introduced.

Response: Responsible gambling has now been spelt out in full.

Writing quality & clarity

The manuscript is mostly well-written. Headings and sub-headings numbering may be helpful for a reader.

Response: We have not included this numbering as this is not in the journal formatting guidelines.

A few items which may require clearer wording:
Lines 72-73: The four subscales were not introduced prior that.

Response: We have now clarified that these were four subscales of the Positive Play Scale.

Lines 77-84: Terms low-risk and non-problem gambling appear to be used interchangeably which should not be the case.

Response: We have now used “lower-risk” consistently in this paragraph.

Lines 85-89: This part appears to belong to methods section.

Response: We agree that this refers to the methods, but we have left it here as we believe that using a method that avoids the confounding issues referred to in the introduction is also part of the rationale for the study.

Reference list

See comment on Introduction regarding the need of wider literature overview.

Response: We have now included a wider range of literature.

There is an error in a doi number in Wood et al. (2017) (line 395).

Response: We have now corrected this.
