# Supplementary material for: Avoiding gambling harm: An evidence-based set of safe gambling practices for consumers
Source: PLoS One. 2019 Oct 17;14(10):e0224083. doi: 10.1371/journal.pone.0224083 (PMC6797237; doi:10.1371/journal.pone.0224083)
Supplement: Peer review history

# Peer review: Decision Letter 0

**DOI:** 10.1371/journal.pone.0224083.r001

**Authors:** Simone Rodda

21 Aug 2019

PONE-D-19-19226

Avoiding gambling harm: An evidence-based set of safe gambling practices for consumers

PLOS ONE

Dear Professor Hing,

Thank you for submitting your manuscript to PLOS ONE. After careful consideration, we feel that it has merit but does not fully meet PLOS ONE’s publication criteria as it currently stands. Therefore, we invite you to submit a revised version of the manuscript that addresses the points raised during the review process.

The reviewers are very positive towards your manuscript and the main issue they would like addressed is the introduction.

We would appreciate receiving your revised manuscript by Oct 05 2019 11:59PM. When you are ready to submit your revision, log on to https://www.editorialmanager.com/pone/ and select the 'Submissions Needing Revision' folder to locate your manuscript file.

If you would like to make changes to your financial disclosure, please include your updated statement in your cover letter.

To enhance the reproducibility of your results, we recommend that if applicable you deposit your laboratory protocols in protocols.io, where a protocol can be assigned its own identifier (DOI) such that it can be cited independently in the future. For instructions see: http://journals.plos.org/plosone/s/submission-guidelines#loc-laboratory-protocols

Please include the following items when submitting your revised manuscript:

- A rebuttal letter that responds to each point raised by the academic editor and reviewer(s). This letter should be uploaded as separate file and labeled 'Response to Reviewers'.
- A marked-up copy of your manuscript that highlights changes made to the original version. This file should be uploaded as separate file and labeled 'Revised Manuscript with Track Changes'.
- An unmarked version of your revised paper without tracked changes. This file should be uploaded as separate file and labeled 'Manuscript'.

Please note while forming your response, if your article is accepted, you may have the opportunity to make the peer review history publicly available. The record will include editor decision letters (with reviews) and your responses to reviewer comments. If eligible, we will contact you to opt in or out.

We look forward to receiving your revised manuscript.

Kind regards,

Simone Rodda

Academic Editor

PLOS ONE

Journal Requirements:

1. When submitting your revision, we need you to address these additional requirements.

Please ensure that your manuscript meets PLOS ONE's style requirements, including those for file naming. The PLOS ONE style templates can be found at

http://www.journals.plos.org/plosone/s/file?id=wjVg/PLOSOne_formatting_sample_main_body.pdf and http://www.journals.plos.org/plosone/s/file?id=ba62/PLOSOne_formatting_sample_title_authors_affiliations.pdf

2. Please provide additional details regarding participant consent. In the ethics statement in the Methods and online submission information, please ensure that you have specified (1) whether consent was informed and (2) what type you obtained (for instance, written or verbal, and if verbal, how it was documented and witnessed).

3. We note that you have indicated that data from this study are available upon request. PLOS only allows data to be available upon request if there are legal or ethical restrictions on sharing data publicly. For information on unacceptable data access restrictions, please see http://journals.plos.org/plosone/s/data-availability#loc-unacceptable-data-access-restrictions.

In your revised cover letter, please address the following prompts:

a) If there are ethical or legal restrictions on sharing a de-identified data set, please explain them in detail (e.g., data contain potentially identifying or sensitive patient information) and who has imposed them (e.g., an ethics committee). Please also provide contact information for a data access committee, ethics committee, or other institutional body to which data requests may be sent.

b) If there are no restrictions, please upload the minimal anonymized data set necessary to replicate your study findings as either Supporting Information files or to a stable, public repository and provide us with the relevant URLs, DOIs, or accession numbers. Please see http://www.bmj.com/content/340/bmj.c181.long for guidelines on how to de-identify and prepare clinical data for publication. For a list of acceptable repositories, please see http://journals.plos.org/plosone/s/data-availability#loc-recommended-repositories.

We will update your Data Availability statement on your behalf to reflect the information you provide.

[Note: HTML markup is below. Please do not edit.]

Reviewers' comments:

Reviewer's Responses to Questions

**Comments to the Author**

1. Is the manuscript technically sound, and do the data support the conclusions?

The manuscript must describe a technically sound piece of scientific research with data that supports the conclusions. Experiments must have been conducted rigorously, with appropriate controls, replication, and sample sizes. The conclusions must be drawn appropriately based on the data presented. 

Reviewer #1: Partly

Reviewer #2: Yes

**********

2. Has the statistical analysis been performed appropriately and rigorously? 

Reviewer #1: I Don't Know

Reviewer #2: I Don't Know

**********

3. Have the authors made all data underlying the findings in their manuscript fully available?

The PLOS Data policy requires authors to make all data underlying the findings described in their manuscript fully available without restriction, with rare exception (please refer to the Data Availability Statement in the manuscript PDF file). The data should be provided as part of the manuscript or its supporting information, or deposited to a public repository. For example, in addition to summary statistics, the data points behind means, medians and variance measures should be available. If there are restrictions on publicly sharing data—e.g. participant privacy or use of data from a third party—those must be specified.

Reviewer #1: No

Reviewer #2: Yes

**********

4. Is the manuscript presented in an intelligible fashion and written in standard English?

PLOS ONE does not copyedit accepted manuscripts, so the language in submitted articles must be clear, correct, and unambiguous. Any typographical or grammatical errors should be corrected at revision, so please note any specific errors here.

Reviewer #1: Yes

Reviewer #2: Yes

**********

5. Review Comments to the Author

Please use the space provided to explain your answers to the questions above. You may also include additional comments for the author, including concerns about dual publication, research ethics, or publication ethics. (Please upload your review as an attachment if it exceeds 20,000 characters)

Reviewer #1: PONE-D-19-19226

Avoiding gambling harm: An evidence-based set of safe gambling practices for consumers

Thank you for the opportunity to review the above manuscript. The manuscript presents results of an online survey which identified gambling behaviour practices predicting a reduction of harm or increase in gambling harm. The result is a proposed set of 9 evidence-based gambling practices indicated to support safe gambling for consumers.

There are a number of strengths to the current study, including addressing an important gap in the current literature for evidence-based support of gamblers in reducing gambling behaviour, the considered analytic techniques used and the use of a specific, targeted sample. Overall, the paper was well written and presented, clear aims that were met with appropriate methodology and analysis, and interpretation of how results could be utilised in the applied setting.

I have a number of comments and queries for the authors to consider. Given the limitations of the market sample recruitment (to be detailed), lack of representativeness suggested, and the somewhat arbitrary selection of SPGs, I suggest that there are particular aspects that need to be addressed prior to wide dissemination and application of this particular set of 9 SPGs. In particular, more details and/or justification regarding the selection of the 9 SPGs based on the results and the imbalance between the 9 that reduce (practices to do n=6) and those that increase (practices to not do, n=3).

Abstract

Strong summary of paper, albeit not including the 9 SPGs.

Introduction

Good introduction to paper. Important to note that strategies are being recommended, advertised and used by professionals and consumers alike, with limited or no evidence of effectiveness. The aim is therefore an important one to address.

Method

- Additional details required to explain the approach taken for identifying potential SGPs; the literature review (e.g., search terms, scope) and content analysis (e.g., consumer practices and/or professional recommendations); actual use vs recommended use, etc. The authors indicate that details are reported elsewhere (deidentified for submission purposes), making it difficult to review these processes here.

- How did the research/treatment/training/policy professional group determine helpfulness of the each identified practice? Professional judgement or other evidence-based, local, unpublished, grey literature data perhaps? Could these professionals add additional practices, or was there an indication that the list was exhaustive? Rating scale and cut-off for low, resulting in discarded practices, not detailed, nor how differences between group decided.

- Participants were to have engaged in ‘at least monthly gambling’ – over what period of time? Presuming 12 months, given that the response to practices was whether used within past 12 months, but not clear?

- Total number of items in survey and estimated duration for completion

- Proportion of people commencing and completing survey – missing data?

Results

- I was not familiar with the specific form of regression used, elastic net regression prior to reviewing this paper, and therefore appreciated the details provided. Were there specific assumptions that were tested and met prior to analysis?

- The selection of the final 9 SGPs requires some more clarification. Although as indicated by the authors, selection was somewhat arbitrary (line 254), I am not clear as to why the emphasis is on reducing gambling a(6 items) compared to increased gambling harm (3 items). Also, why stronger performing SGPs were not included. For example, “I often talk about gambling with my friends and/or family” (0.46) and “As a rule, I don’t gamble in the company of an adult who I am the primary carer for, or who is my primary carer (0.36) are stronger than SGPs 5 and 6 which are included. I would be interested in seeing perhaps the top 5 performing for reducing gambling and the top 5 performing for increased gambling harm, and then those selected to form the “most evidently important SGPs”. I suggest that understanding this selection process more fully is required. I appreciate that some SPGs were selected as more readily translatable, however by excluding a focus on some items, those working with individuals may miss important details (such as those with an adult carer, for example). Presenting the top performing SPGs is a missed step, particularly given the emphasis on the evidence-based approach to identifying strategies.

- Potential limitations as distinctions between, for example, Having a budget for gambling vs sticking to the budget for gambling. How was this considered?

- SPG 8 – 34.5% while text reports 65% (line 268-269) – should read ‘did not use’ (as done in first phrase of sentence, not latter)

- Table 4 heading to include N or sub-set of gamblers in title

- Table 6 – for consistency, don’t to be do not

Discussion

- Well considered potential application of the SPGs broadly, however, as with other work examining the use of strategies, this study does not indicate how gamblers should implement such recommended strategies. Aspects of when, how, with who, etc are not detailed, making appropriate application of the strategy more difficult perhaps. Reference to mechanisms such as implementation intentions, action and coping planning etc. could support those wishing to action such SPGs.

- Did the authors wish to comment on the strategies that were not predicting increase/reduction of harm that they would have anticipated would have been relevant or are reported as popularly used but not predictive; particularly given all items included had been indicated as helpful by the professional group?

- Were strategies/practices identified in more recent work, included in the initial pool of practices (unable to determine from manuscript details), or have some potentially effective practices recently identified (e.g., in work by Rodda and colleagues) been missed due to timing and could be considered in future? How do the effective practices sit within the different types/groups of change strategies identified? For example, I would also be interested in a note regarding the timing of the SPGs; that is, those that are enacted pre-gambling, and others while gambling, or those specific to gambling and not (e.g., keeping a household budget).

o Rodda, S. N., Bagot, K.L., Cheetham, A., Hodgins, D. C., Hing, N., & Lubman, D. I. (2018). Types of change strategies for limiting or reducing gambling behaviours and their perceived helpfulness: A factor analysis. Psychology of Addictive Behaviors, 32(6), 679-688.

o Rodda, S.N., Bagot, K.L., Manning, V., & Lubman D.I. (2019). ‘Only take the money you want to lose’ strategies for sticking to limits in electronics venues, International Gambling Studies, pp1-19. Published on line 24 May 2019.

o Rodda, S.N., Bagot, K.L., Manning, V., & Lubman D.I. (2019). “It was terrible. I didn’t set a limit”. Proximal and distal prevention strategies for reducing the risk of a bust in gambling venues. Journal of Gambling Studies, pp1-15. Published on line, 29 January 2019.

- As the initial sources of practices were both academic (literature review) and presumably applied (web site content), a note as to the source of the effective practices may be warranted. This inclusion would allow an indication as to whether web sources are an appropriate avenue for gamblers to locate effective strategies. As it is likely that individuals seeking to change their behaviour would be able to readily access on-line sources, this is an important note to include.

- Finally, given the heteregenous group of gambling behaviours of the selected sample, how these practices may or may not apply across different types of gambling may be considered by future evaluation.

Congratulations to the authors on this work which I expect to be of great interest to those working and researching in this area.

Reviewer #2: This manuscript reports on the development of a 9-item guideline of safe gambling practices (SGPs) which can be used for reducing gambling harms among gamblers and also for estimating prevalence of safe gambling on a population level for public health purposes. This manuscript, extends the existing body of knowledge by looking into the strategies’ relationship to gambling harms and by making focus on proactive strategies. The study had three aims: 1) to identify SGPs that best predict non-harmful gambling; 2) examine their frequency; 3) examine the relationship between SPGs use and gambler characteristics. The study recruited a convenience sample of 1,174 gamblers. Analysis utilised elastic net regressions to increase predictability of the models. The authors reported nine SGPs which were most effective for reduction of gambling harms. The dual use of the guidelines – as an intervention for gambling harm reduction and as a public health tool for informing gambling policy, - is particularly interesting. Overall, the manuscript is of interest for health professionals and researchers in the gambling field. Some minor corrections may improve the readability.

Abstract and introduction

The abstract provides good summary of the study. However, the meaning of the opening sentence can be clarified as it is not clear how ‘infrequent’ use relates to the strategies being ‘protective’ (line 25-26).

Introduction explains the background of this study well and provides good justification for it. However, a wider use of literature would make the argument stronger. The whole introduction is based on only 9 publications. In-text citations can be more precise as not all statements are supported by a reference (e.g., lines 51 and 54) which makes it difficult for a reader to check the manuscript’s claims.

The rational of the study is clear, but summarising it at the end of the introduction before the aims would make for a good presentation.

Figures and tables

There are six tables in this manuscript. The numbering of the tables should follow the order of appearing in text. However, Table 4 is referenced straight after Table 1 (line 124). Table 2 and Table 6 do not appear to be tables to me. Consider presenting the items from these tables as bulleted text, not a table. Table 3 formatting is not consistent, e.g., ‘Income’ needs to be in italics and sub-items need to be indented. Also consider merging Tables 4 and 5 and presenting frequencies for the ‘remaining SGPs’.

Methods

The manuscript presents a sound well-designed study appropriate for meeting the aims of this research. The limitations of the modest sample size are discussed in ‘Limitations’ and are not of a concern.

Please consider reporting the study period more precisely, e.g., month (line 108). Also consider explaining the incentives to the participants a bit more (line 109) for replication purposes.

Results, discussion, conclusions

Results of the study support the conclusions drawn by the authors. The results contribute to a better understanding of behavioural strategies used for gambling harm reduction and provide empirical evidence of their effectiveness.

Line 269: The proportion of people who used this strategy is indicated 65% while Table 5 reports 34.5%. My understanding is that the confusion comes from the wording of this sentence.

Line 279 and onwards in ‘Results’ section: Please check what symbol to use for reporting sample means. The manuscript uses capitalised M. Also consider reporting statistics for means, e.g., standard deviations or confidence intervals.

Lines 295-301: The manuscript reports frequencies of gambling activities of low prevalence only, while the reader may be also interested in the frequencies of more prevalent gambling activities in relation to SGP score.

Line 328: This sentence creates an impression that the findings of this study will be applicable only in Alberta. It appears to me that these findings can inform harm minimisation efforts across wider locations.

Lines 332-334: Please consider rephrasing this sentence for better clarity.

Line 347: Abbreviation RG was not introduced.

Writing quality & clarity

The manuscript is mostly well-written. Headings and sub-headings numbering may be helpful for a reader.

A few items which may require clearer wording:

Lines 72-73: The four subscales were not introduced prior that.

Lines 77-84: Terms low-risk and non-problem gambling appear to be used interchangeably which should not be the case.

Lines 85-89: This part appears to belong to methods section.

Reference list

See comment on Introduction regarding the need of wider literature overview.

There is an error in a doi number in Wood et al. (2017) (line 395).

**********

6. PLOS authors have the option to publish the peer review history of their article (what does this mean?). If published, this will include your full peer review and any attached files.

If you choose “no”, your identity will remain anonymous but your review may still be made public.

**Do you want your identity to be public for this peer review?** For information about this choice, including consent withdrawal, please see our Privacy Policy.

Reviewer #1: No

Reviewer #2: No

[NOTE: If reviewer comments were submitted as an attachment file, they will be attached to this email and accessible via the submission site. Please log into your account, locate the manuscript record, and check for the action link "View Attachments". If this link does not appear, there are no attachment files to be viewed.]

While revising your submission, please upload your figure files to the Preflight Analysis and Conversion Engine (PACE) digital diagnostic tool, https://pacev2.apexcovantage.com/. PACE helps ensure that figures meet PLOS requirements. To use PACE, you must first register as a user. Registration is free. Then, login and navigate to the UPLOAD tab, where you will find detailed instructions on how to use the tool. If you encounter any issues or have any questions when using PACE, please email us at figures@plos.org. Please note that Supporting Information files do not need this step.

# Author comment: Author response to Decision Letter 0

**DOI:** 10.1371/journal.pone.0224083.r002

1 Oct 2019

Please see the attached file 'Response to Reviewers'

Attachment Submitted filename: Response to Reviewers.docx Click here for additional data file.

# Editor report: Decision Letter 1

**DOI:** 10.1371/journal.pone.0224083.r003

**Authors:** Simone Rodda

7 Oct 2019

Avoiding gambling harm: An evidence-based set of safe gambling practices for consumers

PONE-D-19-19226R1

Dear Dr. Hing,

We are pleased to inform you that your manuscript has been judged scientifically suitable for publication and will be formally accepted for publication once it complies with all outstanding technical requirements.

Within one week, you will receive an e-mail containing information on the amendments required prior to publication. When all required modifications have been addressed, you will receive a formal acceptance letter and your manuscript will proceed to our production department and be scheduled for publication.

Shortly after the formal acceptance letter is sent, an invoice for payment will follow. To ensure an efficient production and billing process, please log into Editorial Manager at https://www.editorialmanager.com/pone/, click the "Update My Information" link at the top of the page, and update your user information. If you have any billing related questions, please contact our Author Billing department directly at authorbilling@plos.org.

If your institution or institutions have a press office, please notify them about your upcoming paper to enable them to help maximize its impact. If they will be preparing press materials for this manuscript, you must inform our press team as soon as possible and no later than 48 hours after receiving the formal acceptance. Your manuscript will remain under strict press embargo until 2 pm Eastern Time on the date of publication. For more information, please contact onepress@plos.org.

With kind regards,

Simone Rodda

Academic Editor

PLOS ONE

Additional Editor Comments (optional):

Reviewers' comments:

# Editor report: Acceptance letter

**DOI:** 10.1371/journal.pone.0224083.r004

**Authors:** Simone Rodda

9 Oct 2019

PONE-D-19-19226R1 

Avoiding gambling harm: An evidence-based set of safe gambling practices for consumers 

Dear Dr. Hing:

I am pleased to inform you that your manuscript has been deemed suitable for publication in PLOS ONE. Congratulations! Your manuscript is now with our production department. 

If your institution or institutions have a press office, please notify them about your upcoming paper at this point, to enable them to help maximize its impact. If they will be preparing press materials for this manuscript, please inform our press team within the next 48 hours. Your manuscript will remain under strict press embargo until 2 pm Eastern Time on the date of publication. For more information please contact onepress@plos.org.

For any other questions or concerns, please email plosone@plos.org. 

Thank you for submitting your work to PLOS ONE.

With kind regards,

PLOS ONE Editorial Office Staff

on behalf of

Dr. Simone Rodda 

Academic Editor

PLOS ONE
